# Whole Genome Sequencing, Focused Assays and Functional Studies Increasing Understanding in Cryptic Inherited Retinal Dystrophies

**DOI:** 10.3390/ijms23073905

**Published:** 2022-03-31

**Authors:** Benjamin M. Nash, Alan Ma, Gladys Ho, Elizabeth Farnsworth, Andre E. Minoche, Mark J. Cowley, Christopher Barnett, Janine M. Smith, To Ha Loi, Karen Wong, Luke St Heaps, Dale Wright, Marcel E. Dinger, Bruce Bennetts, John R. Grigg, Robyn V. Jamieson

**Affiliations:** 1 Eye Genetics Research Unit, Sydney Children’s Hospitals Network, Save Sight Institute, Children’s Medical Research Institute, University of Sydney, Sydney, NSW 2000, Australia; benjamin.nash@health.nsw.gov.au (B.M.N.); alan.ma@health.nsw.gov.au (A.M.); tloi@cmri.org.au (T.H.L.); john.grigg@sydney.edu.au (J.R.G.); 2 Specialty of Genomic Medicine, Faculty of Medicine and Health, University of Sydney, Sydney, NSW 2000, Australia; gladys.ho@health.nsw.gov.au (G.H.); elizabeth.farnsworth@health.nsw.gov.au (E.F.); janine.smith@health.nsw.gov.au (J.M.S.); luke.stheaps@health.nsw.gov.au (L.S.H.); dale.wright@health.nsw.gov.au (D.W.); bruce.bennetts@health.nsw.gov.au (B.B.); 3 Sydney Genome Diagnostics, Western Sydney Genetics Program, Sydney Children’s Hospitals Network, Westmead, NSW 2145, Australia; karen.wong1@health.nsw.gov.au; 4 Western Sydney Genetics Program, Department of Clinical Genetics, Sydney Children’s Hospitals Network, Westmead, NSW 2145, Australia; 5 Kinghorn Centre for Clinical Genomics, Garvan Institute of Medical Research, Darlinghurst, NSW 2010, Australia; a.minoche@garvan.org.au (A.E.M.); mcowley@ccia.org.au (M.J.C.); 6 Lowy Cancer Research Centre, Children’s Cancer Institute, University of New South Wales, Randwick, NSW 2031, Australia; 7 St Vincent’s Clinical School, Faculty of Medicine, University of New South Wales, Randwick, NSW 2031, Australia; 8 South Australian Clinical Genetics Service, Women’s and Children’s Hospital, North Adelaide, SA 5006, Australia; christopher.barnett@sa.gov.au; 9 Faculty of Health and Medical Sciences, University of Adelaide, Adelaide, SA 5005, Australia; 10 School of Biotechnology and Biomolecular Sciences, Faculty of Science, University of New South Wales, Randwick, NSW 2031, Australia; m.dinger@unsw.edu.au; 11 Department of Ophthalmology, The Children’s Hospital at Westmead, Sydney Children’s Hospitals Network, Westmead, NSW 2145, Australia; 12 Save Sight Institute and Specialty of Ophthalmology, Faculty of Medicine and Health, University of Sydney, Sydney, NSW 2000, Australia

**Keywords:** inherited retinal dystrophy, whole genome sequencing, gene panels, RNA analysis

## Abstract

The inherited retinal dystrophies (IRDs) are a clinically and genetically complex group of disorders primarily affecting the rod and cone photoreceptors or other retinal neuronal layers, with emerging therapies heralding the need for accurate molecular diagnosis. Targeted capture and panel-based strategies examining the partial or full exome deliver molecular diagnoses in many IRD families tested. However, approximately one in three families remain unsolved and unable to obtain personalised recurrence risk or access to new clinical trials or therapy. In this study, we investigated whole genome sequencing (WGS), focused assays and functional studies to assist with unsolved IRD cases and facilitate integration of these approaches to a broad molecular diagnostic clinical service. The WGS approach identified variants not covered or underinvestigated by targeted capture panel-based clinical testing strategies in six families. This included structural variants, with notable benefit of the WGS approach in repetitive regions demonstrated by a family with a hybrid gene and hemizygous missense variant involving the opsin genes, *OPN1LW* and *OPN1MW*. There was also benefit in investigation of the repetitive GC-rich ORF15 region of *RPGR*. Further molecular investigations were facilitated by focused assays in these regions. Deep intronic variants were identified in *IQCB1* and *ABCA4*, with functional RNA based studies of the *IQCB1* variant revealing activation of a cryptic splice acceptor site. While targeted capture panel-based methods are successful in achieving an efficient molecular diagnosis in a proportion of cases, this study highlights the additional benefit and clinical value that may be derived from WGS, focused assays and functional genomics in the highly heterogeneous IRDs.

## 1. Introduction

The inherited retinal dystrophies (IRDs) are collectively responsible for impaired vision in more than five million people worldwide, with genomic population data suggesting an estimated prevalence of at least one in 1400 [1]. The degree and kind of vision defect varies depending on the IRD subtype, and is often progressive with severity usually increasing with age. IRDs collectively are the most common cause of legal blindness amongst the working-age population [2,3]. IRDs are classified into broad groups based on the photoreceptor cell type affected, such as cone and cone-rod dystrophy (CD/CORD), retinitis pigmentosa (RP) and generalized retinal dystrophy (RD) [4]. Autosomal dominant, autosomal recessive, X-linked and mitochondrial forms of inheritance are all observed, and cases may present initially with an isolated retinal presentation (non-syndromic) or be part of a larger syndromic presentation with extraocular features. Ophthalmic presentations can often be subtle and there can be considerable overlap in clinical diagnoses. There is a very high degree of genetic heterogeneity amongst the IRDs, with over 300 genes and loci known to be implicated in disease (RetNet: https://sph.uth.edu/retnet/, accessed on 11 February 2022). Further complexities exist with single genes contributing to several types of IRD or variant types in a gene which may lead to disease in autosomal dominant or autosomal recessive forms, such as *PRPH2* [OMIM: 179605] and *PROM1* [OMIM: 604365]. These factors all lead to challenges in obtaining an accurate genetic diagnosis, which is essential for access to emerging genetic therapies which require a specific genetic diagnosis for clinical trial or therapy eligibility [5,6,7].

The implementation of next-generation sequencing (NGS) technologies in research and diagnostic genomic laboratories worldwide over the last decade has enabled the examination of genetically complex disease such as the IRDs to become more accessible and efficacious in the clinical setting [8]. There are several large IRD cohort studies which have demonstrated this through the application of various genomic strategies using targeted capture of custom designed IRD gene panels or the examination of in silico gene panels applied to partial exome (clinical exome, CES), whole exome (WES) or whole genome (WGS) sequencing approaches [9,10,11,12]. These have yielded varying diagnostic rates between 55–75%, which is consistent with that observed in our own clinical laboratory testing. Furthermore, the application of WGS has been shown to increase diagnostic yield due to the superior ability to detect and resolve structural variants (SVs) and to interrogate GC-rich sequences and deep intronic regions, compared with WES and other targeted capture approaches [11].

There is an increasing reliance on clear genomic diagnosis in ophthalmology, with clinical therapies and trials becoming increasingly available for IRD patients and where genetic diagnostic certainty is required for patients to benefit from the new and emerging therapy options. In this study we describe six IRD families previously unsolved using routine clinical exome or other diagnostic testing in whom we noted an advantage when we applied a WGS approach for variant examination followed by focused assays and functional studies in specific cases.

## 2. Results

### 2.1. Molecular Diagnoses Obtained in Previously Unsolved IRD Families

WGS was performed to investigate genomic variants in six families that were previously unsolved on targeted capture or panel based sequencing from partial exome or WES or other clinically available testing at the time of their initial review. This was in the context of a research study involving 100 unrelated IRD cases, where WGS was performed in 39 cases, CES in 37, and 24 having both. An overall molecular diagnosis was achieved in 62 cases and in 6 of these (6/100) WGS proved valuable, providing a ~10% uplift (6/62 solved cases) in diagnostic yield, including variants located in highly homologous and/or GC rich regions (two families), deep intronic variants [+/− 50bp from canonical splice site boundary] (two families), and other copy number variants (SV/CNVs) (two families).

### 2.2. WGS Analysis Revealed Complex Hybrid Opsin SV and Missense Allele in Repetitive Region

**Figure 1:**
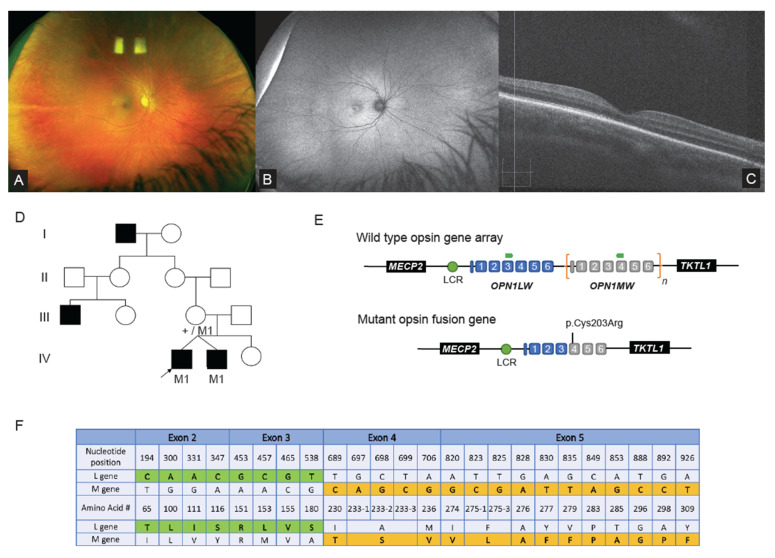
WGS and focused assays identify opsin gene hybrid involving within chromosome Xq28 (**A**) Ultra-wide-field pseudo colour fundus images from the proband shows a normal appearance. (**B**) Wide-field fundus autofluorescence (UWF-FAF) showed some irregular hyperautofluorescence at the fovea with the remaining fundus having a normal WF-FAF pattern. (**C**) OCT assessment shows focal ellipsoid zone outer retinal discontinuity; (**D**) Pedigree analysis across four generations, suggestive of X-linked inheritance (pedigree abbreviated for illustrative purposes), the proband and twin sibling both had visual acuities of 6/60 in each eye, M1 = mutant hybrid opsin allele, + = wild type allele; (**E**) Illustration of the genomic arrangement of the tandem opsin genes within chromosome Xq28. Wild type and the complex allele identified in Family 1 are shown, with primer locations for the focused assay indicated by green arrows; (**F**) Table highlighting the differences in single nucleotide (c.) and amino acid (p.) sequence between the *OPN1L*W (NM_020061) and *OPN1MW* (NM_000513.2) genes (Table modified from source [13]). Unique coding *OPN1LW* sequence detected in our family highlighted in green, and *OPN1MW* coding sequence is highlighted in orange/yellow (LCR: Locus Control Region). OCT Optical Coherence tomography, UWF-FAF Ultra-wide field fundus autofluorescence.

In Family 1, a large non-consanguineous family of middle eastern ethnicity presented with nystagmus for ophthalmic and clinical genomic review. Initial clinical ophthalmic examination of the proband (Figure 1A–D) revealed high hypermetropia (+8.0 diopters each eye), and a normal retinal and macular appearance (Figure 1A). The paediatric visual electrophysiology protocol was tolerated for a subset of the International Society for Clinical Electrophysiology of Vision (ISCEV) standard full field electroretinogram (ffERG). The results showed loss of photopic responses for the LA3.0 with preserved scotopic responses (DA0.01 and DA3.0). Other stimuli were not tolerated. At age 11, ultra-wide field fundus autofluorescence (UWF-FAF) (Optos plc, Dunfermline, UK) revealed hyperautofluorescence involving the fovea (Figure 1B) in one individual. Optical coherence tomography (OCT) (Zeiss Meditec Cirrus OCT, Dublin, CA, USA) showed focal ellipsoid zone outer retinal discontinuity (Figure 1C). A clinical diagnosis of cone dystrophy was made and pedigree analysis revealed several males affected with a similar phenotype across four generations, suggestive of an X-linked inherited condition (Figure 1D). Prior to this study *RPGR* was interrogated with Sanger sequencing with no likely causative variant identified.

WGS was performed on the proband, his affected twin and their mother, with initial variant calling of IRD gene lists finding no causative variants. Subsequent repeat ISCEV standard ffERG (Espion Diagnosys, Lowell, MA, USA) at age 11 years in the proband, identified a preserved scotopic ffERG and an absence in the photopic stimuli (LA3.0 and 30 hz flicker) consistent with a diagnosis of either achromatopsia or blue cone monochromatism (BCM). Given the clinical suspicion of BCM, manual interrogation of the *OPN1LW* and *OPN1MW* opsin gene read data in IGV took place and was suggestive of a structural variant, with a reduction in the read depth when available data from the two affected males in the family were compared to unaffected male WGS data of the same loci (Supplementary Figure S1). A previously reported pathogenic variant in *OPN1MW*(NM_000513.2):c.607T>C p.(Cys203Arg) was also detected in all available reads, which has been reported in association with a single 5′ *OPN1LW*, 3′ *OPN1MW* hybrid gene [14].

To confirm the WGS findings, the unique coding regions of *OPN1LW* and *OPN1MW* were amplified using published primers, as in previous studies [15]. This showed the *OPN1MW*(NM_000513.2):c.607T>C p.(Cys203Arg) together with a sequencing pattern specific for *OPN1LW* for exon 2 and 3, and *OPN1MW* for exons 4 and 5 (as exons 1 and 6 do not contain transcript unique variants) (Figure 1E,F). To confirm the presence of the complex hybrid opsin gene a focused assay was designed with primers specific to exon 3 of *OPN1LW* coding sequence and reverse primer specific to exon 4 *OPN1MW* coding sequence, allowing for the detection of the hybrid opsin gene allele using standard PCR protocols. Segregation studies of available samples with the focused assay confirmed affected males and obligate female carriers in the family (Figure 1D; individuals III-3, IV-1 & IV-2).

### 2.3. Interrogation of WGS Data Identifies an RPGR ORF15 Variant, in a Repetitive GC Rich Region

**Figure 2:**
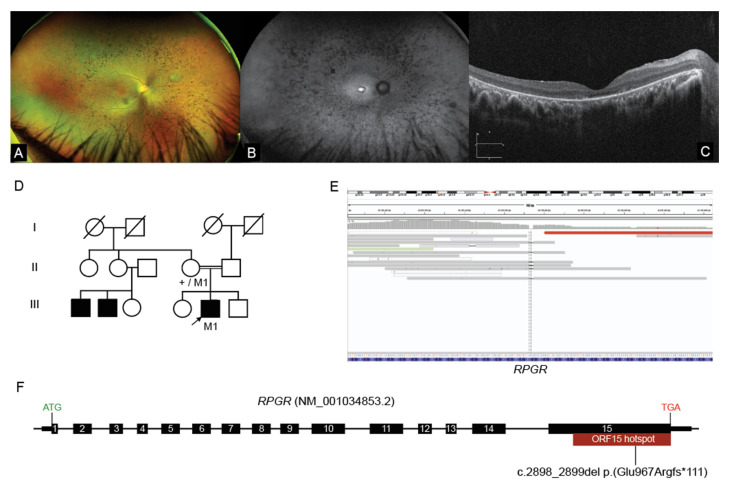
WGS identifies loss of function variant within the *RPGR* mutation hotspot ORF15 (**A**) Ultra-wide-field pseudo colour fundus images showing widespread retinal pigment migration in a “bone spicule” pattern with associated outer retinal atrophy; (**B**) UWF-FAF showing a central area hyper autofluorescence surrounded by a ring of incomplete hypo autofluorescence. Beyond the vascular arcades there are speckled areas of hypo autofluorescence of varying density; (**C**) OCT assessment shows preservation of an attenuated ellipsoid zone (EZ) and outer retinal structures at the fovea. In the para macular regions there is significant attenuation to loss of the EZ line representing outer retinal structures. (**D**) Pedigree of Family 2, M1 = *RPGR* ORF15 mutant allele, + = wild type allele; (**E**) IGV screenshot showing coverage of the *RPGR*(NM_001034853.2):c.2898_2899del p.(Glu967Argfs * 111) variant detected on WGS (dashed vertical lines), located within the highly repetitive GC-rich ORF15 locus; (**F**) Illustration of the *RPGR* ORF15 gene structure depicting the location of the variant identified within the low complexity region of the ORF15 mutation hotspot. OCT Optical Coherence tomography, UWF-FAF Ultra-wide field fundus autofluorescence, EZ ellipsoid zone. WGS identifies loss of function variant within the *RPGR* mutation hotspot ORF15 (**A**) Ultra-wide-field pseudo colour fundus images showing widespread retinal pigment migration in a “bone spicule” pattern with associated outer retinal atrophy; (**B**) UWF-FAF showing a central area hyper autofluorescence surrounded by a ring of incomplete hypo autofluorescence. Beyond the vascular arcades there are speckled areas of hypo autofluorescence of varying density; (**C**) OCT assessment shows preservation of an attenuated ellipsoid zone (EZ) and outer retinal structures at the fovea. In the para macular regions there is significant attenuation to loss of the EZ line representing outer retinal structures. (**D**) Pedigree of Family 2, M1 = *RPGR* ORF15 mutant allele, + = wild type allele; (**E**) IGV screenshot showing coverage of the *RPGR*(NM_001034853.2):c.2898_2899del p.(Glu967Argfs * 111) variant detected on WGS (dashed vertical lines), located within the highly repetitive GC-rich ORF15 locus; (**F**) Illustration of the *RPGR* ORF15 gene structure depicting the location of the variant identified within the low complexity region of the ORF15 mutation hotspot. OCT Optical Coherence tomography, UWF-FAF Ultra-wide field fundus autofluorescence, EZ ellipsoid zone.

A 14-year-old male proband of middle eastern ethnicity presented for ophthalmic and genetics review in Family 2. Night vision difficulties and visual field restriction were noted in the proband from eight years of age. Best corrected visual acuity was noted as 6/18−1 and 6/15+2, respectively. Ultra-wide field (UWF) pseudocolour fundus images showed equatorial intraretinal pigment migration in a bone spicular pigmentation pattern (Figure 2A). There was arteriolar attenuation but normal optic discs. There was early macular atrophy. UWF-FAF showed an intense hyper autofluorescence (hyperAF) localized region at the fovea (Figure 2B). OCT showed attenuation and discontinuity of the ellipsoid zone (EZ) in the paramacular region. The subfoveal EZ line was preserved but attenuated (Figure 2C). An ffERG showed undetectable scotopic and photopic responses. These ophthalmic findings were consistent with the diagnosis of retinitis pigmentosa (RP). The parents were of known consanguinity (first degree relatives) and there was a report that other maternal male relatives were affected with RP, leading to a suspicion of possible X-linked inheritance, however these individuals lived overseas and were not available for assessment (Figure 2D).

Initial genomic studies performed using the TruSight One (Illumina, San Diego, CA, USA) targeted capture approach identified no variants of significance in known IRD genes. WGS studies were subsequently undertaken, and given the X-linked family history, a detailed analysis of all available reads across *RPGR* was pursued. This identified a hemizygous 2bp deletion variant within the *RPGR* ORF15 hotspot locus: *RPGR*(NM_001034853.2):c.2898_2899del p.(Glu967Argfs * 111) (Figure 2E). Although the read depth was poor at only 6×, the variant was present in all of the mapped reads. Segregation studies with Sanger sequencing confirmed the presence of the variant and showed that it was maternally carried. The c.2898_2899del variant was classified as pathogenic given it was a loss of function allele located within the ORF15 hotspot locus, absent from the control database gnomAD and had multiple Likely Pathogenic entries in ClinVar. On subsequent review of the read data of the previous targeted capture sequencing on this patient, it was evident that the variant was not detected, as it was located in a coverage gap due to the highly repetitive GC-rich complex region of the *RPGR* ORF15 locus (Figure 2F). The coverage limitation impacting routine targeted capture sequencing approaches of *RPGR* ORF15 was further evident upon examining read data from other unrelated male and female samples, demonstrating the additional value of using a focused analysis of WGS data in this family.

### 2.4. Deep Intronic Variant Identified on WGS Shown on RNA Studies to Create an IQCB1 Pseudoexon

**Figure 3:**
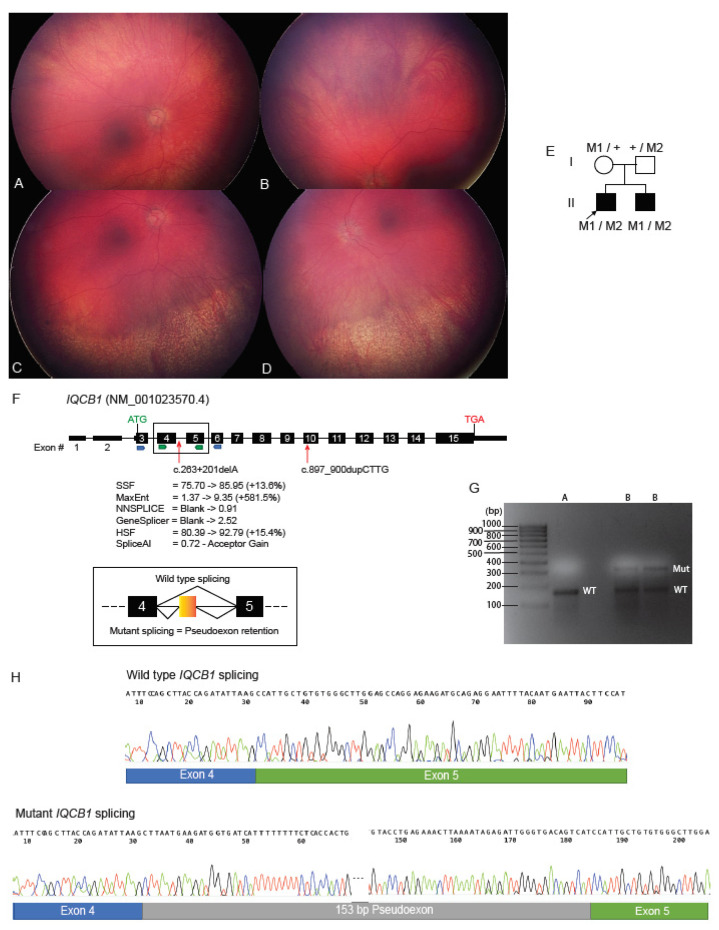
WGS and RNA studies identify a novel deep intronic *IQCB1* variant which activates a pseudoexon. (**A**) Right superior fundus, (**B**) Left superior fundus, (**C**) Right inferior fundus, and (**D**) Left inferior fundus shown for proband II.1 with widefield Retcam fundus images (RetCam, Natus, Pleasanton, CA, USA). The main clinical changes are seen in the inferior fundus of each eye (**C**,**D**). There is retinal atrophy with granular pigmentary changes involving the inferior fundus in each eye. There are also fine speckled pigmentary changes in the superior fundus on clinical exam, which is difficult to appreciate in the Retcam photos. (**E**) Pedigree of Family 3, M1 = *IQCB1*:c.897_900CTTGdup, M2 = *IQCB1*:c.263 + 201delA, + = wild type; (**F**) Illustration of *IQCB1* gene structure, with exon numbering and the location of the variants identified in this study indicated by red arrows. Splicing prediction algorithm data for the novel intronic variant is shown, suggesting the activation of a cryptic acceptor splice site. Coloured arrows underneath indicate primer pair locations in exons 3 to 6 and 4 to 5. Orange box illustrates the location of the pseudoexon introduced between exons 4 and 5; (**G**) Agarose gel image showing the presence of the wild type band [WT, 161bp] in both the control (**A**) and proband (**B**), with the larger sized mutant band [Mut, 314bp] also present in the proband samples; (**H**) Sanger sequencing traces showing the retention of 153bp of intron 4 sequence into the mutant band of *IQCB1* RNA. Note: only flanking 5 and 3′ ends of the pseudoexon are pictured and not all 153bp.

Family 3 was of Caucasian ethnicity and consisted of two affected male siblings who presented for ophthalmic and genetics review due to nystagmus and poor vision within the first decade of life. There was no known consanguinity or family history of retinal dystrophy. At the age of three years, fundal examination of the older sibling (proband) showed small optic discs and no pigmentary retinopathy. ERG studies were consistent with a generalized retinal disorder, with extinguished cone and rod responses. Limited visual evoked potential (VEP) studies at this time raised the possibility of optic nerve or cortical visual dysfunction, and subsequent brain MRI imaging showed mild periventricular white matter changes. Difficulties with social interactions and repetitive stereotypical behaviours were noted, however a formal behavioural assessment that demonstrated this did not satisfy the criteria for autism spectrum disorder. The younger male sibling was first examined at the age of two years with nystagmus and poor vision noted from infancy. Fundus features included inferior retinal atrophy with granular pigmentary changes in both the left (Figure 3A,C) and right eyes (Figure 3B,D). ERG studies showed markedly reduced rod and cone responses. There was no renal phenotype evident in either of the affected brothers. The ophthalmic findings were consistent with Leber Congenital Amaurosis (LCA) in both siblings.

Initial commercial studies in the proband of a panel of LCA genes showed a single heterozygous 4 base pair duplication: *IQCB1*(NM_001023570.4):c.897_900CTTGdup p.(Ile301Leufs * 42). Homozygous and compound heterozygous variants in the *IQCB1* gene have been associated with LCA with or without the renal phenotype of nephronophthisis [16]. The 4 base pair duplication was present in low frequency in the gnomAD control database at a frequency of 0.00006, and has also been reported in the literature as pathogenic in compound heterozygosity in individuals affected with LCA plus extraocular renal manifestations [11,17]. Given that the single heterozygous variant in *IQCB1* identified alone was insufficient to cause the phenotype, we suspected there may be a cryptic variant on the other allele contributing to the phenotype. To investigate this, both affected siblings and parental samples underwent WGS to facilitate segregation and allow for guided analysis of deep intronic or other regulatory variants. The c.897_900CTTGdup variant was shown to be maternally inherited, and was also present in the affected sibling. Examination of the remaining paternally inherited genomic *IQCB1* variants common among the affected siblings identified a candidate deep intronic variant c.263 + 201delA. This variant was novel and was absent from gnomAD. Examination of a potential effect on splicing using Alamut Visual and SpliceAI suggested that the c.263 + 201delA variant created a cryptic acceptor site at the adjacent nucleotide position c.263 + 203 (Figure 3F).

To examine the molecular consequence of the novel deep intronic variant further, studies on RNA isolated from a skin sample biopsy of the proband were undertaken. Conversion of total RNA to cDNA took place, followed by amplification of the *IQCB1* sequence between exons 3 and 6 and exons 4 and 5, which both showed the presence of two sized alleles when resolved on agarose gel (Figure 3G). The isolation and sequencing of these bands showed that the genomic variant was activating a cryptic acceptor site at position c.263 + 203 with a subsequent cryptic donor site being used at position c.263 + 356, describable as *IQCB1*(NM_001023570.4):r.263_264ins263 + 203_263 + 356. This resulted in the retention of 153bp of intronic *IQCB1* sequence introducing a pseudoexon in the reading frame between exons 4 and 5, predicted to introduce 23 abnormal amino acids followed by a premature stop codon and hence a truncated protein of only 111 amino acids, describable as p.(His89Leufs * 23) (Figure 3H).

### 2.5. ABCA4 Deep Intronic Variant Identified on WGS

A 46-year-old male proband of Caucasian background from Family 4 presented for genetics review with an initial ophthalmic diagnosis of retinal dystrophy with Stargardt-like features. There was no known consanguinity or family history of retinal dystrophy. Visual acuity was noted as 6/9 bilaterally. Fundoscopy examinations over the previous 15 years showed a progressive macular dystrophy with grey subretinal flecks throughout the posterior pole surrounding the disc and the macula, with developing patches of macular atrophy and sparing of the foveal tissue. Subsequent examination at 51 years showed irregular atrophy affecting both maculas extending outside the arcades, with no signs of a bull’s eye macular atrophy or retinal flecks. Visual acuity changes to 6/24 and 6/19, respectively, were also noted, with evidence of early bilateral cataracts.

Initial clinical testing using the TruSight One (Illumina, San Diego, CA, USA) targeted capture approach identified a single heterozygous previously reported pathogenic variant, namely *ABCA4*(NM_000350.2): c.4577C>T p.(Thr1526Met), which is an established pathogenic allele reported in literature in association with Stargardt disease (https://www.ncbi.nlm.nih.gov/clinvar/variation/99303/, accessed on 11 February 2022) [18]. WGS studies were sought to investigate a second disease causing allele and identified a heterozygous variant in *ABCA4*(NM_000350.2):c.5196 + 1137G>A. The deep intronic variant c.5196 + 1137G>A was also classified as pathogenic and has also been reported in the literature to activate a cryptic splice acceptor site, and retention of a pseudoexon [19]. Review of the data from previous diagnostic testing revealed that this variant was located in a genomic region which was not captured in the TruSight One (Illumina, San Diego, CA, USA) library preparation from that time, therefore not providing coverage of this region during previous variant curation. It is noteworthy however, that frequently reported deep intronic disease alleles are now often routinely examined as they are included in the capture of whole exome sequencing panels, such as the Agilent SureSelect Clinical Research Exome V2 (Agilent Technologies, Santa Clara, CA, USA).

### 2.6. WGS Identifies Homozygous KCNV2 Deletion

**Figure 4:**
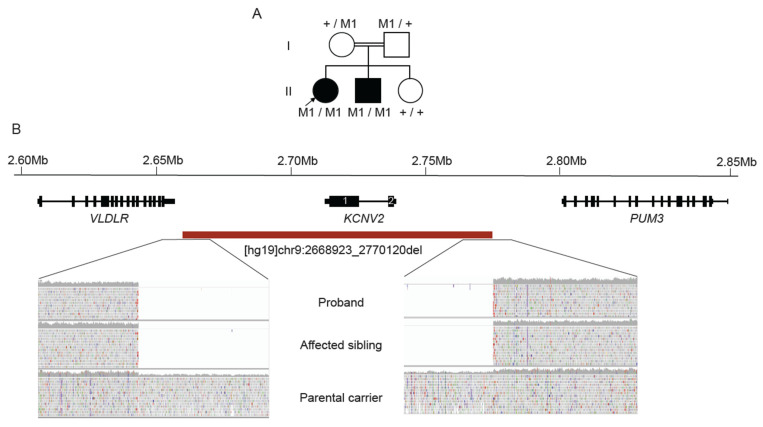
Homozygous deletion identified involving *KCNV2*. (**A**) Pedigree of family with two siblings with cone dystrophy, suggestive of autosomal recessive inheritance. M1 = *KCNV2* heterozygous deletion allele, + = wild type allele; (**B**) Location of the genomic deletion identified by WGS including IGV screenshots of reads spanning the 5′ and 3′ breakpoints. Proband (II.1) [top] & affected sibling (II.2) [middle] showing no reads (homozygous deletion). Parental carrier (I.1) [bottom] showing reduced read depth (heterozygous deletion) compared to flanking adjacent genomic segments.

Family 5 was of Middle Eastern ethnicity, with an eight year old female and her five year old brother presented for ophthalmic and genetics review (Figure 4A). The parents were known to be consanguineous and there was no family history of retinal dystrophy. The female proband was referred at age 5 ½ years with reduced vision not able to be corrected with glasses. The initial assessment identified 6/19 visual acuity in each eye. The vision deteriorated over the next 12 months. Color vision assessment was performed using both the City University color vision test and the Ishihara color vision test. The City university test was normal. There was a mild protan defect on the Ishihara color vision test. Clinically there was no nystagmus, normal cycloplegic refraction and the fundus examination was reported as within normal limits. A retinal dystrophy was suspected. Electrophysiology suggested reduced amplitude cone responses and lesser attenuation of rod responses, suggesting a cone or cone-rod dystrophy. The younger male sibling had visual acuity of Right 6/19 and Left 6/15 with latent nystagmus and a moderate left exotropia. Fundoscopy examination was within normal clinical limits. Ophthalmic examination of the parents and the youngest sibling were normal. Given the suggestive autosomal recessive cone or cone rod dystrophy pedigree, clinical diagnostic testing of the *ABCA4* gene was arranged at the time, which was uninformative.

WGS identified a novel 101Kb homozygous deletion involving the *KCNV2* gene, [hg19]chr9:2668923_2770120del (Figure 4B). This finding was confirmed using SNP-CMA (Supplementary Figure S2), with segregation studies showing both parents to be heterozygous carriers and the affected sibling also being homozygous for the deletion. While there are three heterozygous deletions with unique breakpoints involving the whole *KCNV2* gene listed in the population database gnomAD, the 101Kb deletion seen in the present family was not previously reported. Biallelic pathogenic variants involving the *KCNV2* gene have been associated with cone dystrophy with supernormal rod electroretinogram (CDSRR), which is an autosomal recessive disorder [20]. Further ophthalmic review will hopefully determine if the affected individuals may have this clinical phenotype. The *KCNV2* gene is 2168bp in size spanning 2 exons, encoding a voltage gated potassium channel (Kv8.2) which maintains the photoreceptor membrane potential [20]. Homozygous and compound heterozygous mutations including genomic deletions of variable size spanning the *KCNV2* gene have been detected using CMA or targeted capture sequencing approaches, and previously reported in the literature as pathogenic [21,22].

### 2.7. WGS Identifies a Single Exon CNV in ABCA4

**Figure 5:**
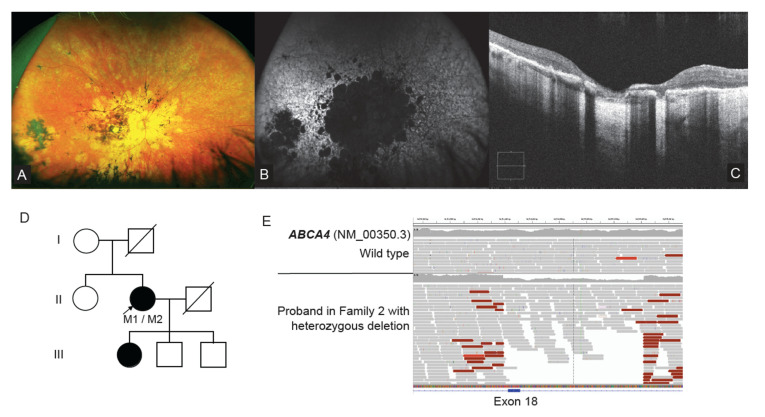
WGS resolves single exon CNV in *ABCA4*. (**A**) Ultra-Wide field pseudo colour fundus images show significant atrophy involving the posterior pole in each eye. There was blotchy retinal atrophy extending to the equator in each eye; (**B**) UWF-FAF shows dense loss of autofluorescence in the posterior pole of both eyes extending beyond the vascular arcades with coalescing paving stone like areas of dense hypo autofluorescence. In the periphery there is patchy areas of hyper and hypo autofluorescence; (**C**) OCT showed almost complete loss of the outer retinal layers at the macular with significant disruption and loss of the EZ in the para macular region; (**D**) Pedigree of Family 6. M1 = *ABCA4*:c.5461-10T>C, M2 = *ABCA4* exon 18 deletion; (**E**) IGV screenshot of the 1029bp deletion involving *ABCA4* exon 18, plus flanking intronic segments. Genomic deletion is describable as [hg19]chr1:94514389_94515418del. OCT Optical Coherence tomography, UWF-FAF Ultra-wide field fundus autofluorescence, EZ ellipsoid zone.

In Family 6, a 45-year-old female proband presented for ophthalmological and genetics review with longstanding vision loss. Ophthalmic investigations showed an advanced stage retinal dystrophy. Her visual acuity was hand movements only in each eye. Fundal examination showed significant atrophy involving the posterior pole in each eye (Figure 5A). There was patchy retinal atrophy extending to the equator in each eye. Wide-field autofluorescence (WF-FAF) imaging showed loss of autofluorescence in the posterior pole of both eyes extending beyond the vascular arcades with coalescing paving stone like areas of dense hypo autofluorescence (Figure 5B). In the periphery there were patchy areas of hyper and hypo autofluorescence. OCT showed almost complete loss of the outer retinal layers at the macular with significant disruption and loss of the ellipsoid zone in the para macular region (Figure 5C). These findings were consistent with an ophthalmic diagnosis of advanced Stargardt disease or cone rod dystrophy. There was no known consanguinity, and the proband’s daughter was also reported to be experiencing difficulties with her vision (Figure 5D).

Previous clinical testing using CES examining IRD disease genes identified a single heterozygous change *ABCA4*(NM_000350.2):c.5461-10T>C. Proband WGS research testing was available at the time of review and examination of Stargardt and macular dystrophy genes by WGS identified single exon heterozygous 1029bp deletion involving exon 18 of *ABCA4*, in addition to the pathogenic variant c.5461-10T>C (Figure 5E). Orthogonal high resolution CGH-CMA studies confirmed the presence of exon 18 deletion (Supplementary Figure S3), and segregation studies are planned to determine the biallelic phase of the variants when family samples become available (Figure 5D). This copy number variant was not called in the initial targeted capture sequencing due to the variability in read depth coverage at this genomic position. Given this limitation, it remains clear that orthogonal assays are still required to reliably call and validate CNVs in targeted capture sequencing—highlighting further the power of using the WGS strategy.

## 3. Discussion

In this study we have successfully demonstrated the effectiveness of using WGS to provide molecular diagnoses in cases which were, or would have been, difficult to solve using targeted or full exon capture-based approaches. This success was due to the ability of WGS to more reliably detect SV/CNVs, especially in repetitive regions such as the *OPN1LW* and *OPN1MW* region, as well variants in repetitive GC-rich regions such as *RPGR* ORF15, while also allowing for the interrogation of deep intronic sequences—all potentially cryptic in an exon capture-based approach. This study demonstrates the benefit of incorporating functional genomic techniques in determining novel variant pathogenicity, with RNA studies determining the molecular consequence of a novel deep intronic variant which activates a pseudoexon in the gene *IQCB1*. We also emphasize the need for careful interpretation of WGS data and congruent focused assays in some contexts, such as interrogation of the opsin genes, which allowed for the detection of a pathogenic hybrid opsin gene.

There is a revolution in ophthalmic genomics with the rapid emergence and application of gene therapies for various disorders, such as Luxturna gene therapy for patients with biallelic pathological variants in *RPE65* [5]. Any possibility of gene therapy for a patient, however, is reliant on accurate diagnostic genomics. While there have been enormous advances in genomics over the last decade, there remains more work to be done to increase diagnostic rates among the IRDs, with rates varying between 25–45% of families yet to be solved in various IRD subtypes [9,10,11,12]. These findings are suggestive of (1) the lack of suitable approaches to identify all the variants in known disease genes implicated in IRD, (2) disease genes yet to be identified in these conditions, being as high as 20% of the disease loci mapped in CD/CORD (RetNet: https://sph.uth.edu/retnet/sum-dis.htm#A-genes, accessed on 11 February 2022), (3) the potential non-Mendelian or multigenic inheritance, and (4) the involvement of novel genetic mechanisms yet to be described. Applying focused assays in concert with WGS and functional studies, as applied in this study, are becoming fruitful approaches to filling in the known limitations of clinical testing and increasing the diagnostic yield. In addition, the discovery of novel disease genes and the expansion of genotype-phenotype correlations in already described disease genes, as in our previous studies, will also increase the diagnostic yield [23,24].

There are multiple IRD disease genes which are unable to be fully interrogated using targeted capture based sequencing approaches. Significant disease associated loci such as the repetitive GC-rich *RPGR* ORF15 region and the highly homologous opsin gene array within chromosome Xq28 are currently poorly covered in CES and WES strategies alone, requiring additional separate focused assays for analysis [13,25,26]. Moreover, there are other IRD disease genes where a significant proportion of pathogenic alleles lie within intronic or non-coding regions such as *ABCA4* [27] and the *PRDM13* associated North Carolina Macular Dystrophy locus [28]. As in this study, carefully focused curation of appropriate genomic regions and variants can aid in improving diagnostic rates moving forward. Consideration of deep phenotyping information pointing to a particular gene or region of interest, or focus on the second allele in autosomal recessive disease, helped focus analysis of WGS data to reveal variants in repetitive and/or GC rich regions (Families 1 and 2), deep intronic variants (Families 3 and 4), and small SV/CNVs (Families 5 and 6).

This is the first study to report the finding of a hybrid opsin gene detected using WGS. The widely used approach involves a specific assay of a long range PCR of the opsin gene array, followed by amplification of multiple nested amplicons specific to the *OPN1LW* or *OPN1MW* gene loci to facilitate their sequence analysis [26]. In the present case, initial ophthalmic information suggested a cone dystrophy, hence investigation of *RPGR* was pursued and WGS was undertaken with the aim of potentially discovering a novel disease gene. Subsequent detailed ophthalmic phenotype information directed the analysis specifically to the complex *OPN1LW* or *OPN1MW* disease loci and led to the manual visualisation of WGS read data and identification of the hemizygous disease causing allele. This specific hybrid gene and missense variant genotype has been described in a number of patients with BCM [26]. The p.(Cys203Arg) has been shown to result in a structurally defective protein and therefore the protein product expected from the hybrid gene is also non-functional [29]. This case is a worthy example of the need for caution when relying purely on automated variant calling and variant curation, especially in repetitive or pseudogene regions of the genome with low read coverage. This case also emphasizes the flexibility of a WGS strategy, where multiple rounds of focused analysis can be performed on the same raw sequencing dataset, effectively without the need for any further additional wet bench work.

WGS is a superior technology for the interrogation of highly complex, repetitive and GC-rich regions of the genome when compared to targeted capture sequencing methods [30]. The *RPGR* ORF15 hotspot locus is one of these regions, where often an alternative strategy is needed to accurately examine the genomic sequence in detail [25]. The *RPGR* ORF15 variant identified by WGS in Family 2 was initially overlooked by previous targeted capture sequencing due to the inability of the technology to map informative sequences, highlighting the valuable ability of WGS to detect these variants from within a single assay. The further refinement and development of long read sequencing technologies such as Nanopore and PacBio systems further promise to provide the ability to resolve variants in *RPGR* ORF15 and other similar challenging loci; however, these approaches have yet to be translated into routine clinical testing [31]. Studies applying PacBio long-read sequencing to IRDs have specifically demonstrated the potential of this technology, identifying a large disease causing tandem duplication involving *NMNAT1* while also providing variant haplotype-phase information [32].

Interpretation of deep intronic genomic variants >50bp from natural canonical splice site junctions remains challenging. While tools exist to predict a potential effect on splicing, such as the introduction of cryptic donor and acceptor sites in non-coding sequences, these tools are only indictors of a splicing aberration and not considered as standalone evidence in determining pathogenicity [33]. Newer splicing prediction tools such as SpliceAI use different algorithms and interrogate larger windows of genomic sequence to better predict cryptic splice sites in large intronic regions, which is needed with the increased application of WGS in diagnostics [34]. We were able to narrow down the several deep intronic variants called within the *IQCB1* sequencing data in Family 3 to identify the candidate variant with the use of segregation analysis and the various splicing prediction tools within Alamut Visual and SpliceAI. Confirmation of the molecular consequence of this novel variant was still required, and was possible with RNA obtained from a skin puncture biopsy, given that the causative gene *IQCB1* had expression in skin fibroblasts. The gold standard approach, however, to determine the functional consequence of deep intronic variants remains cDNA studies from RNA sourced from relevant tissues. In IRD genes, this approach often remains challenging, due to gene expression of many disease genes being limited to the retina, where a biopsy remains clinically unobtainable. Other tools such as minigene assays and patient derived induced pluripotent stem cells (hiPSC) may also be applied to aid in interpretation [35]. As novel deep intronic variants become increasingly implicated in disease, future work is needed to translate promising technologies such as RNAseq, exon trap assays and hiPSC and/or organoid models from research laboratories into diagnostics in order to streamline the confirmation of pathogenicity in accordance with ACMG guidelines [33].

There are advantages in transition of clinical testing from examining only protein coding regions of the genome (WES) to the whole genome (WGS), as WGS provides the ability to reanalyze essentially the same dataset over time as medical knowledge advances. The discussion of Family 4 highlights this point, as the original clinical testing did not include the genomic region of the pathogenic deep intronic variant in the targeted capture method used. Currently, the inclusion of known pathogenic non-coding alleles, such as those seen in *ABCA4*, are included in an incremental process, relying on commercial providers to include examination of these regions in regularly updated versions of their products. The implementation of WGS strategies in clinical testing will eliminate this time-consuming need of updating the targeted regions captured, allowing for the re-interrogation of these regions in silico using the data from the single WGS wet-bench assay. In this study, we wish to highlight the ability of WGS to resolve cases in instances where the existing targeted capture methods have known limitations. We demonstrate the value of WGS in these situations using a combination of in silico variant calling and manual interrogation, and demonstrate the importance in being able to re-interrogate the same dataset with increasing focus as the diagnostic need requires. However, there are also limitations which remain with the use of WGS. Reliable interrogation of *RPGR* ORF15 and the opsin gene cluster within chromosome Xq28 is still difficult with short-read WGS approaches, and in particular using routine variant calling/filtering pipelines. In addition, there also remain difficulties for largescale implementation of WGS in diagnostic laboratories related to cost and availability of effective bioinformatic data handling and data storage needs, in accordance with local health, ethics and governance requirements. Also, the appropriate consideration of the clinical and laboratory management of reportable secondary findings, such as variants in cancer or cardiac predisposition genes, remains to be fully adopted worldwide [36,37].

Targeted capture, genomic PCR-based approaches have limited ability to detect SV/CNVs, and rely on coverage and an average read depth of exons or regions of interest which are sensitive to lack of uniformity and reproducibility in read depth and quality [38]. WGS is a superior technology in this regard due to the ability to obtain more informative split reads which span the SV/CNV breakpoint, allowing for the precise mapping of breakpoints. However, while promising, limitations remain, and it is evident that further development is required for more accurate SV/CNV calling tools and still relies on visual interrogation of WGS reads in many cases [39]. The contribution of SV/CNVs as disease alleles are significant, and reports in up to 7% have been identified in other IRD cohort studies [40]. The deletion involving *ABCA4* in Family 6 in this study highlights the limitations of targeted capture sequencing technologies given the small size (~1kb) and intronic breakpoints of this single exon deletion. This emphasizes the value of diagnostic application of WGS to facilitate more accurate SV/CNV analysis and will further contribute to increasing molecular diagnostic rates in the IRDs.

## 4. Materials and Methods

### 4.1. Cohort and Patient Ascertainment

Families with a clinical ophthalmic diagnosis of retinal dystrophy and no molecular diagnosis on previous routine clinical diagnostic testing were investigated. These families were recruited from genetic eye clinics within tertiary hospitals in Sydney, Australia. The ethnicity of families recruited was diverse, which is representative of the mixed cultural population of the Greater Western Sydney area. Where available, family samples were also collected for segregation studies, and informed patient consent was obtained. Genomic DNA was extracted from peripheral blood lymphocytes using standard protocols.

### 4.2. Ophthalmic Examination

Clinical ophthalmic examination was performed in all patients. Where possible, ophthalmic imaging was performed. Widefield imaging was obtained using either the Optos California (Optos plc, Dunfermline, UK), encompassing up to 200 degrees, or the Retcam fundus imaging system (RetCam, Natus, Pleasanton, CA, USA). Ultra-wide field fundus autofluorescence was obtained with the Optos system. Macular SD-OCT scans were acquired with the Spectralis SD-OCT (Heidelberg Engineering, Heidelberg, Germany) and/or Cirrus 4000 SD-OCT (Carl Zeiss Meditec, Dublin, CA, USA).

### 4.3. Genomics and Bioinformatics

Whole genome sequencing (WGS) was performed using Illumina TruSeq Nano HT (Illumina, San Diego, CA, USA). Subsequent libraries were sequenced on a HighSeq X Ten (Kinghorn Centre for Clinical Genomics, Garvan Institute of Medical Research, Sydney, Australia) instrument. Reads were aligned to the NCBI build GRCh37 or hg19 reference genome, with variant calling performed according to GATK best practice guidelines (https://software.broadinstitute.org/gatk/best-practices, accessed on 1 January 2022). Structural and copy number variation was called using ClinSV, which examines a combination of read depth, discordant and split read information (https://github.com/KCCG/ClinSV, accessed on 1 January 2022) [41]. Variants identified on WGS were filtered and priortised using Ingenuity Variant Analysis (Qiagen, Redwood City, CA, USA). All variants were filtered and priortised to: (1) exclude those with an allele frequency >1% observed in either the 1000 genomes, gnomAD and NHLBI ESP control databases; and (2) include those predicted to be damaging or likely to affect protein function using in silico prediction tools SIFT, PolyPhen-2 and MutationTaster. Variants of interest were further interrogated with Alamut Visual v2.8.1 (Interactive-Biosoftware, France) to examine variant population allele frequency. In the first tier of variant curation, an initial gene panel restricted to the clinical IRD subtype was applied. If there were no likely causative variants identified, the genes examined were then expanded to the full retinal dystrophy panel, facilitating curation of variants identified in all IRD gene panels. Prioritised variants of clinical interest were classified according to ACMG guidelines [33]. The review of the sequencing reads was performed using an Integrative Genomics Viewer (IGV) to ensure candidate variants of interest were true calls and to visualize the presence of potential SV/CNVs [42].

Orthogonal molecular genomic techniques were used when appropriate to validate and segregate variants of interest and included custom ultra-high resolution (1×1 million features) comparative genomic hybridisation microarrays (CGH-CMA) (Agilent technologies, Santa Clara, CA, USA), Infinium CytoSNP-850K v1.2 BeadChip Single nucleotide polymorphism chromosome microarrays (SNP-CMA) (Illumina, San Diego, CA, USA), and/or Sanger sequencing. Primers for validation and segregation studies were designed using Primer3 and are available on request, with PCR amplicons sequenced at AGRF (Australian Genome Research Facility, Westmead, NSW, Australia).

### 4.4. Genomic Examination of the OPN1LW and OPN1MW Genes

Given the marked homology (~96%) between the coding regions of *OPN1LW* and *OPN1MW* opsin genes located with chromosome X band q28, manual interrogation of the aligned sequencing reads was undertaken using IGV. Bi-directional Sanger sequencing of coding exon 2 to exon 5 was performed using previously reported approaches to confirm and investigate SNVs [15].

Primers were designed to confirm the hybrid opsin gene for this particular family, with a forward primer specific to exon 3 of *OPN1LW* coding sequence [FWD: 5′-GGGAGAGGTGGCTGGTGGTG-3′] and a reverse primer specific to exon 4 of *OPN1MW* coding sequences [RVS: 5′-CACGATGATGCTGAGTGGGG-3′]. The presence of an amplifiable PCR product using these primers allowed for confirmation of the hybrid opsin gene and also for the detection of obligate female carriers.

### 4.5. RNA Studies of Deep Intronic ICQB1 Variant

Fibroblasts were cultured from a skin biopsy from the proband from Family 3 and total RNA was extracted using the RNeasy micro kit (Qiagen, Redwood City, CA, USA). Total RNA was converted to cDNA using the SuperScript IV 1st Strand Synthesis System kit (Invitrogen, Waltham, MA, USA). Primers were designed using Primer3, with forward primers located in exons 3 and 4, with reverse primers located within exons 5 and 6, and sequences are available on request. PCR was performed following standard cycling conditions with amplified products resolved on agarose gel electrophoresis. Both wild type and mutant bands were excised and purified using the Wizard^®^ SV Gel and PCR Clean-Up System (Promega, Madison, MI, USA) before Sanger sequencing (AGRF, Westmea, NSW, Australia).

## 5. Conclusions

While targeted capture panel-based methods are a widely applied and successful strategy in achieving an efficient molecular diagnosis in a proportion of IRD cases, this study has highlighted WGS as a superior technology for detection of variants in repetitive and/or GC rich regions, deep intronic regions or where there are small or complex SVs/CNVs, which together were implicated in 6% of a heterogenous IRD cohort. However, detection of these variants often requires focused, detailed analysis of the WGS data informed by further clinical phenotype or pedigree information. In addition, focused assays, orthogonal approaches and functional studies are frequently required to validate the identified variants. These factors emphasize the need for development of efficient approaches for incorporation of genomic sequence data analyses and functional genomics approaches into the clinical diagnostic workflow.

## Data Availability

All supporting data from this study is available, please contact the corresponding author.

## Supplementary Materials

The following supporting information can be downloaded at: https://www.mdpi.com/article/10.3390/ijms23073905/s1.
