# Supplementary material for: Whole Genome Sequencing, Focused Assays and Functional Studies Increasing Understanding in Cryptic Inherited Retinal Dystrophies
Source: Int J Mol Sci. 2022 Mar 31;23(7):3905. doi: 10.3390/ijms23073905 (PMC8999823; doi:10.3390/ijms23073905)
Supplement: Supplementary file 1 [file ijms-23-03905-s001.zip › ijms-1613960-supplementary.pdf]

Supplementary Materials

Nash et al. 2022

OPN1LW
(NM_020061)

Male hybrid opsin gene carrier

Normal wild type male

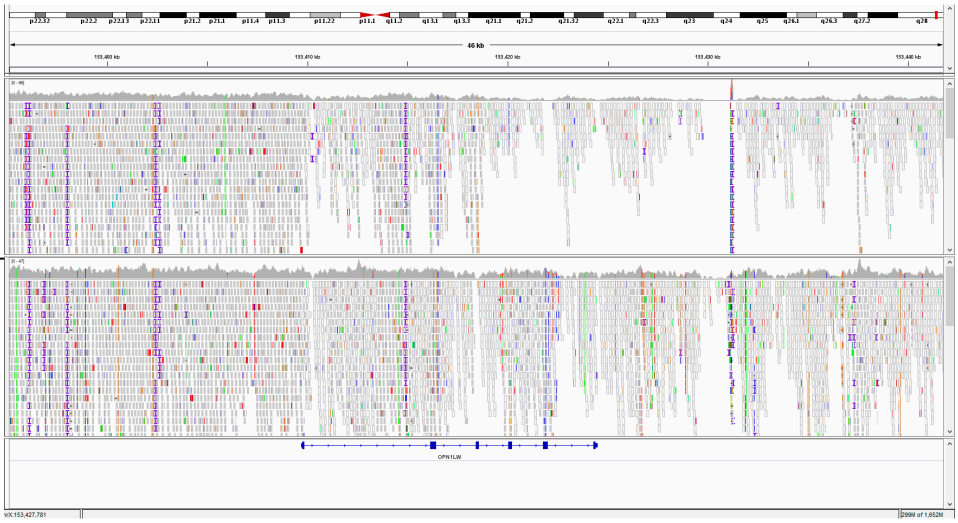

OPN1MW
(NM_000513.2)

Male hybrid opsin gene carrier

Normal wild type male

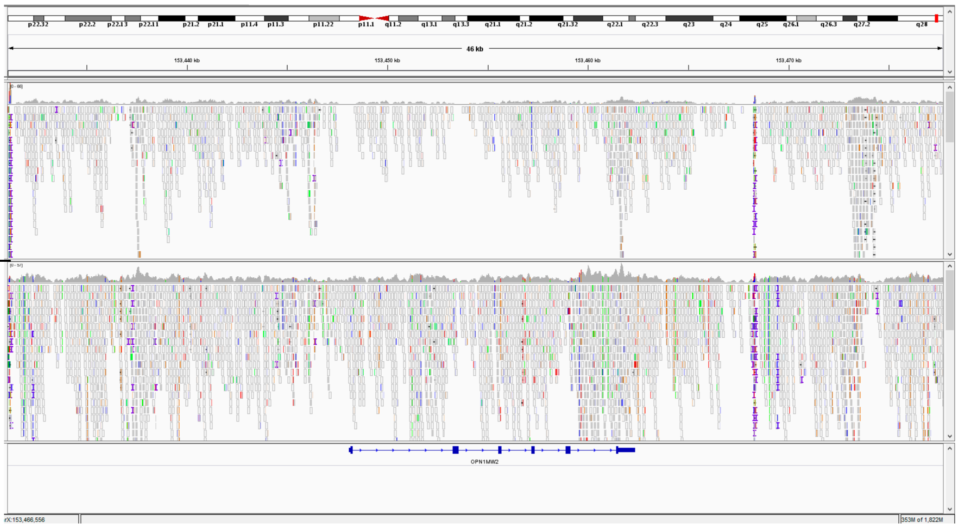

Figure S1. IGV screenshot of read pileup for *OPN1LW* [Top] and *OPN1MW* [Bottom] for both an affected male hemizygous hybrid opsin gene carrier and an unrelated normal wild type male. There was marked reduction in mapped reads noted from *OPN1LW* exon 4 as indicated by read depth, which was suggestive of a structural variant or copy number event.

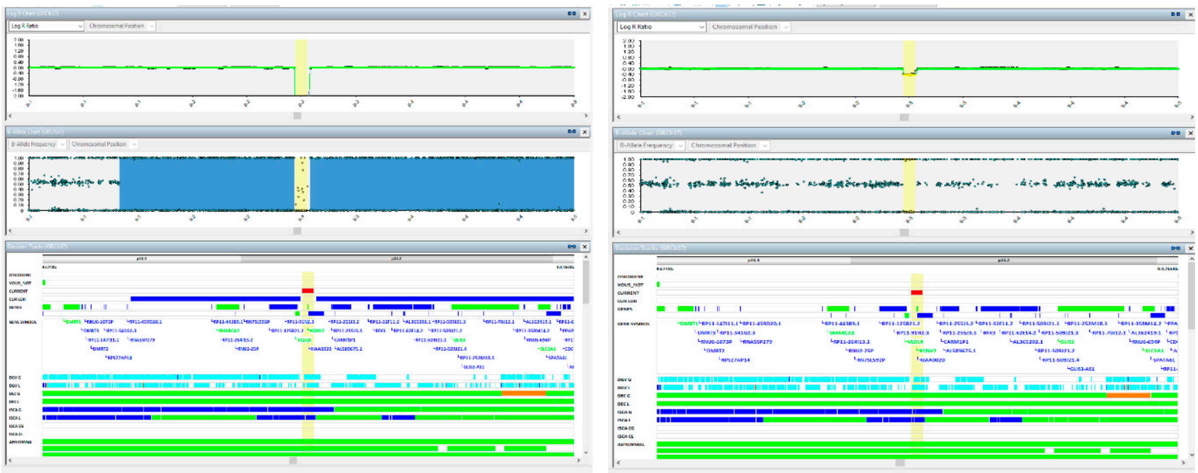

Figure S2. SNP Chromosome microarray (SNP-CMA) data (BlueFuse Multi v4.5) showing the probe shift in LogR and B-allele frequency (BAF) calls consistent with the homozygous deletion [Left] and heterozygous deletion [Right] involving *KCNV2*. Also note that this homozygous deletion lies within a segment with Absence of Heterozygosity (AOH), as indicated by the blue highlighted region in the BAF data [Left].

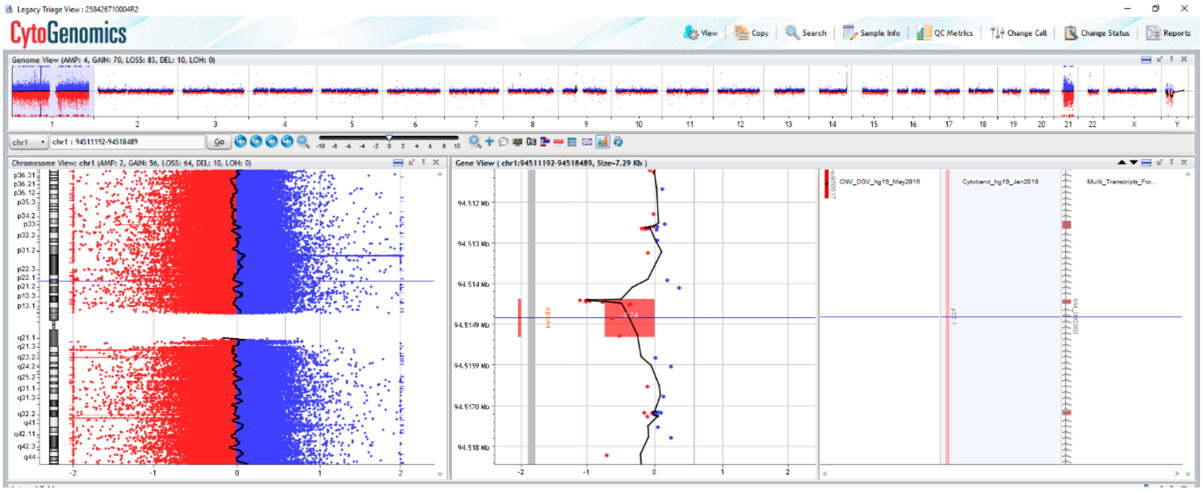

Figure S3. Screenshot of Agilent Cytogenomics software showing CGH-CMA data shifting in Log_2 ratio (-0.74) demonstrating heterozygous deletion involving *ABCA4* exon 18 [highlighted in red].
